# The etiologies of DNA abnormalities in male infertility: An assessment and review

**Published:** 2017-06

**Authors:** Soheila Pourmasumi, Parvin Sabeti, Tahereh Rahiminia, Esmat Mangoli, Nasim Tabibnejad, Ali Reza Talebi

**Affiliations:** *Research and Clinical Center for Infertility, Yazd Reproductive Sciences Institute, Shahid Sadoughi University of Medical Sciences, Yazd, Iran.*

**Keywords:** Sperm chromatin, DNA abnormality, Reactive oxygen species

## Abstract

The sperm DNA damage may occur in testis, genital ducts, and also after ejaculation. Mechanisms altering chromatin remodeling are abortive apoptosis and oxidative stress resulting from reactive oxygen species. Three classifications of intratesticular, post-testicular, and external factors have been correlated with increased levels of human sperm DNA damage which can affect the potential of fertility. Lifestyle, environment, medical, and iatrogenic factors might be considered to cause dysmetabolism to make distracting interactions and endocrine disrupting compounds. As a result, these may induce chromatin/DNA alteration in germ cells, which may be transmitted across generations with phenotypic consequences**. **Alcohol consumption may not increase the rate of sperm residual histones and protamine deficiency; however, it causes an increase in the percentage of spermatozoa with DNA fragmentation and apoptosis**.** In a medical problem as spinal cord injury, poor semen parameters and sperm DNA damage were reported**.** Infection induces reactive oxygen species production, decreases the total antioxidant capacity and sperm DNA fragmentation or antigen production that lead to sperm dysfunctions and DNA fragmentation. While reactive oxygen species generation increases with age, oxidative stress may be responsible for the age-dependent sperm DNA damage. The exposing of reproductive organs in older men to oxidative stress for a long time may produce more DNA-damaged spermatozoa than youngers. Examining the sperm chromatin quality in testicular cancer and Hodgkin’s lymphoma patients prior to chemotherapy demonstrated the high incidence of DNA damage and low compaction in spermatozoa at the time of the diagnosis. In chemotherapy cycle with genotoxic agents in cancer patients, an increase in sperm DNA damage was shown after treatment. In overall, those factors occurring during the prenatal or the adult life alter the distribution of proteins associated with sperm chromatin induce changes in germ cells which can be detected in infertile patients.

## Introduction

Spermatogenesis includes proliferation, meiotic DNA, and differentiation of spermatids into spermatozoa. Due to the complexity and relatively long period of spermatogenesis, the sperm DNA and chromatin may alter at any stage of this process. During spermiogenesis phase, the nuclear histones are replaced at first by transition proteins and then by protamines. The chromatin stability is achieved by disulfide bonds formation during epididymal transit of spermatozoa (1, 2).

It is shown that protamine-induced sperm chromatin condensation is very essential for normal sperm fertility potential and subsequently for normal fertilization and embryonic development (3, 4). There are many studies indicating the elevation of spermatozoa with abnormal chromatin in infertile patientswhen are compared with fertile men (5-7). In addition, there is a negative relationship between sperm chromatin quality and sperm parameters including morphology and motility (8, 9). The presence of sperm with abnormal morphology indicates that spermatozoa cannot complete the process of spermiogenesis (10, 11). The sperm chromatin/ DNA damage may occur in testis, male genital ducts, and also after ejaculation. Modifications in chromatin remodeling, abortive apoptosis, and oxidative stress are three main mechanisms contributing (12-15).

Several etiological factors including intratesticular, post-testicular, and external factors have been correlated with increased levels of human sperm DNA damage which can affect the potential of male fertility. Medical dysfunctions like diabetes mellitus (DM) may have detrimental effects on sperm fertility potential and DNA integrity, also varicocele samples contain a higher proportion of spermatozoa with abnormal DNA and immature chromatin than those from fertile men as well as infertile men without varicocele (16). 

**Figure 1:**
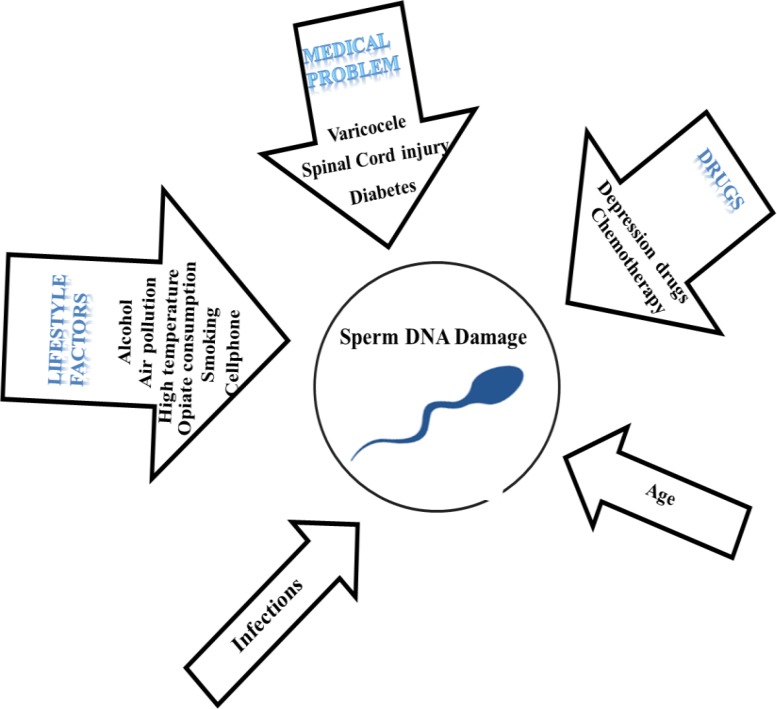
Etiological factors associated with increased human sperm damage

Therefore, varicocele leads to production of spermatozoa with less condensed chromatin and this is one of the potential causes of infertility. In this study, further topics being discussed as well (Figure 1, Table I).

**Table I:** Species and assessment of etiological factors on sperm and male fertility

**Etiology**	**Reference**	**Conclusion**
Diabetes Mellitus		
	Agbaje *et al* (2007)	DM is associated with increased sperm nuclear and mtDNA damages
	Mangoli *et al* (2013)	DM had negative effects on sperm parameters
	Talebi *et al* (2014)	DM may have detrimental effects on sperm fertility potential and DNA integrity
Varicocele		
	Smith *et al* (2006)	Varicocele is associated with high levels of DNA damaged
	Talebi *et al* (2008)	Varicocele samples have a higher percentage of spermatozoa with abnormal DNA
	Ghanaie *et al* (2012)	Varicocele leads to increased sperm DNA damage
	Telli *et al* (2015)	Significant raise in abnormal sperm chromatin in varicocele patients
Spinal cord injury		
	Salsabili *et al* (2009)	SCI is associated with chromatin abnormality
	Mahfouz *et al* (2010)	SCI leads to increase of seminal ROS and sperm DNA fragmentation
	Talebi *et al* (2013)	High level in residual histones and DNA fragmentation in SCI men
Chemotherapy		
	Maselli *et al* (2012)	Chemotherapy changes sperm chromatin integrity
	Paoli *et al* (2015)	Chemotherapy has negative effect on testicular function and spermatogenesis
	Ghezzi *et al* (2016)	Chemotherapy changes chromatin structure of spermatozoa
Infections		
	Huang *et al* (2003)	Infection can lead to modification in genetic component in sperm
	Gallegos *et al* (2008)	Sperm DNA fragmentation in infected patients is higher than non-infected
	Kang *et al* (2012)	HBV infection causes an increase in sperm DNA fragmentation
Depression drugs		
	Koyuncu *et al* (2011)	Psychological drugs affect the sperm count, motility, and morphology
	Khazaie *et al* (201)	Depression drugs have negative effects on the sperm DNA integrity
	Khin *et al* (2015)	Antidepressants can affect on the sperm parameters
Alcohol consumption		
	Talebi *et al* (2011)	Alcohol may cause an increase in percentage of spermatozoa with DNA fragmentation and apoptosis
	La Vignera *et al* (2013)	Alcohol has negative effects on normal sperm parameters
	Komiya *et al* (2014)	Sperm DNA integrity was affected by alcohol
Opiate consumption		
	Song *et al* (2010)	Ecstasy leads to sperm oxidative stress
	Safarinejad *et al* (2013)	Opiate consumers have a significant increase in the quantity of fragmented DNA
Age		
	Wyrobek *et al* (2006)	Young men have a lower percentage of spermatozoa with DNA fragmentation than old men
	Alexeyev *et al* (2009)	Sperm ROS generation increases with age
	Carrell *et al* (2013)	Age has effect on sperm DNA damage and it can be considered as an important etiology of ART failures
Lifestyle		
	Azam *et al* (2003)	Caffeine products can reduce copper and it leads to sperm OS
	Kort *et al* (2006)	Obese patients have higher sperm DNA fragmentation in their ejaculates
	Tamburrino *et al* (2012)	Obesity has negative effects on the sperm DNA integrity
	Meeker *et al* (2010)	Polychlorinated biphenyls can impair the sperm parameters
	Hamad *et al* (2014)	Smoking has negative effects on the sperm chromatin
	Eftekhar *et al* (2016)	Cigarettes that produce reactive oxygen.
Air pollution		
	Boggia *et a*l (2009)	Air pollution is implicated in poor sperm quality
	Ji G *et al* (2010)	Air pollution can induce polymorphisms of sperm genes
	Calogero *et al* (2011)	Sperm parameters were significantly different in motorway tollgate workers

DM: Diabetes mellitus

SCI: Spinal cord injury

HBV:

ROS: Reactive oxygen species

**Diabetes mellitus (DM)**DM is one of the most common diseases that threats human health in the modern world (17). Its effects on human health are mostly due to the hyperglycaemic and hypoinsulinemic states caused by the disease, usually affecting neurological, endocrinological, and reproductive functions (18-20). DM has been related to the sexual dysfunction, both in men and women. It is supposed that neuropathy, vascular deficiency, and psychological problems may be implicated in the pathogenesis of some phenomenon, for example, impotency and ejaculation disorders (21, 22).

Miralles-Garcia *et al* found that insulin-dependent diabetes is associated to decrease in ejaculated semen, vitality and motility of the spermatozoa, but no change in sperm viscosity (23). Defects in insulin secretion may change testicular and accessory sexual glands function. Usually, the concentration of seminal insulin is higher than that in the serum. Numerous studies have demonstrated a marked reduction in sperm quality in diabetic male human and animals (20, 24). Mangoli *et al* showed that DM had negative effects on the sperm parameters and chromatin features in mice (25). also, Talebi *et al* showed that although, the DM may have detrimental effects on the sperm fertility potential and DNA integrity, but, vitamin C, as a potent antioxidant, has positive effects on sperm parameters, sperm function and also prevents sperm chromatin abnormalities and apoptosis in experimentally-induced diabetic mice (26).

Various mechanisms have been proposed for sperm nuclear DNA anomalies. One of them which isis the most important mechanism in the pathogenesis of DM is oxidative stress. However, there are many studies about reactive oxygen species (ROS) production in diabetic patients and beneficial effects of antioxidants (27-30). Agbaje *et al* showed that DM is associated with increased sperm nuclear and mtDNA damages measured by COMET assay and PCR, respectively, and they may impair the reproductive capability of these men (28). Also, they demonstrated significantly increased levels of 8OHdG (ROS-induced DNA damage marker) in spermatozoa of diabetic patients in comparison with non-diabetics. The results of another study showed a significant increase in sperm nuclear DNA fragmentation compared to their non-diabetic counterparts, which is imputable to oxidative stress, resulting in a decrease in blastocyst formation and pregnancy rates and increase in miscarriage rates were well-known in the diabetic group. Additional, variation in mitochondrial DNA seen in DM is reliable for the bad changes observed in motility of spermatozoa (31) (Table I).

**Varicocele**

Varicocele is found in approximately 15% of fertile men and 19-41% of infertile patients. There are several pathways for varicocele-induced infertility (32). One of the main factors responsible for infertility or subfertility in patients with varicocele is sperm DNA damage. It was reported that the rate of apoptotic spermatozoa in patients with varicocele is higher than in those without varicocele (33, 34). Another possible reason in varicocele-associated cases is the elevated intra-testicular temperature during spermiogenesis that increases the sensitivity of sperm DNA to denaturation and high levels of seminal oxidative stress that could impair chromatin packaging (35).

Talebi *et al* examined the effects of varicocele on chromatin condensation and DNA integrity of ejaculated spermatozoa with cytochemical tests (16). The results showed that the varicocele samples have a higher percentage of spermatozoa with abnormal DNA and immature chromatin than fertile men and infertile men without varicocele (16). Smith *et al* showed that the varicocele is associated with high levels of DNA-damaged spermatozoa even in the presence of normal semen profile (36). The results also showed that oxidative stress is related to sperm DNA damage in these patients (36). Also, Enciso *et al* in their study using sperm chromatin dispersion (SCD) test showed that in infertile men with varicocele there is a high relative proportion of sperm cells with extreme levels of nuclear damage (37). 

Studies showed that there is a significant raise in abnormal sperm chromatin condensation in infertile men with varicocele that will noticeably improve following varicocelectomy (38-40). Werthman* et al* examined patients who had preoperative levels of >30% of sperm DNA fragmentation index and exhibited a subsequent significant decrease of the percent of DNA fragmentation index values after surgery (32). Ghanaie* et al* demonstrated that varicocele repair increases the chance of spontaneous pregnancies and live births (41). On the other hand, in an animal model, it is shown that varicocele leads to increased sperm DNA damage and the damage will be decreased by varicocelectomy (42) (Table I).

**Spinal cord injury**

Spinal cord injury (SCI) is a medical problem with arising 1000 new cases per year (43). Approximately more than 80% of these patients are men in reproductive age (44, 45). After SCI, some sexual problem such as impaired erection and ejaculation are happened as well as abnormal semen parameters. It was shown that 25% of SCI men have decreased spermatogenesis both in early and late phases of SCI (46). It is known that sperm motility, morphology and viability significantly decreased in SCI subjects, but, sperm count has remained in the normal range in some studies (47-49). In addition to poor semen parameters, notable sperm DNA damage was reported in both SCI men and experimental animals (45, 50, 51). DNA fragmentation index is seen at a higher rate compared to fertile men (50). This fact is of great importance because DNA integrity is necessary for sperm-oocyte interaction, transmission of genetic information and consequent implantation and embryo cleavage (12, 34). It should be noted that some studies have revealed the importance of DNA fragmentation indices are more important than sperm parameters for prediction of male fertility (52, 53).

As it is shown, spermatozoa with poor DNA integrity may lead to fertilization failure, delayed embryo development, and early pregnancy loss (54, 55). Evenson and Wixon reported that pregnancy rate significantly decreased with semen samples containing more than 30% spermatozoa with fragmented DNA (56). It is well-known that in the cases of SCI, leukocytospermia, lack of ejaculation and seminal fluid stasis, testicular hyperthermia, histologic abnormalities, genitourinary tract infections, and hormonal dysfunction can produce high amounts of ROS (57-63).

The evidence shows that each 25% increase of seminal ROS induces 10% increase in sperm DNA fragmentation (64). Therefore, high levels of ROS and abortive apoptosis are two main causes of sperm chromatin/DNA damage in SCI men (43). Regard to sperm chromatin condensation, Talebi *et al* showed that there is a clear relationship between the percentage of spermatozoa with residual histones and DNA fragmentation in SCI men. They also applied the Chromomycin A3 (CMA3) test and found a remarkable percentage of CMA3 positive sperm cells in the SCI group and concluded that SCI has a negative effect on the sperm protamination. Furthermore, they used the Toluidine Blue (TB) and Aniline Blue staining and reported increased sperm DNA fragmentation and abnormal chromatin condensation as well as high DNA denaturation respectively (65). Similarly, Salsabili *et al* used AB test and found low sperm chromatin condensation and chromatin stability among SCI patients (47). (Table I)

**Cancer and chemotherapy**

Human sperm chromatin is a complex structure; susceptible to damage from different sources including testicular cancer, Hodgkin’s disease, and leukemia which are the most common malignancies affecting men in reproductive age (66-69). 

Several models of cancer therapies have significantly improved survival rates for young patients with some types of malignancies. However, cancer therapies are frequently aggressive, and their side effects are common. Chemotherapy and radiotherapy negatively have an effect on testicular function, spermatogenesis, and sex hormones especially in young men (70, 71). Testicular cancer is a curable malignancy in young men and unilateral orchiectomy is the primary treatment. The majority of young patients with recently diagnosed testicular cancer are worried about future fertility and want to be informed about the influence of different treatment on spermatogenesis (70). Fossa *et al* in their study showed that testicular cancer patients have the high percentage of spermatozoa with abnormal chromatin structure after orchiectomy (72). 

Maselli *et al* demonstrate in rat testicular cancer that exposure to Bleomycin, Etoposide, and Cis-Platinum changes sperm chromatin integrity and sperm head protein profile, but the persistent damage remains in the chromatin structure of spermatozoa (73). O'Flaherty *et al* examined sperm chromatin quality in testicular cancer and Hodgkin’s lymphoma patients prior to chemotherapy, they presented the high incidence of DNA damage and low compaction in spermatozoa from these patients at the time of the diagnosis (74). In another chemotherapy cycle with genotoxic agents in cancer patients, it was shown an increase in sperm DNA damage after treatment (75). 

Tanrikut *et al* also demonstrated that, paroxetine can induce unusual sperm DNA fragmentation in men with normal semen parameters, but they didn’t identify a quantifiable effect of paroxetine on semen parameters (76). The combination of the comet assay and tests that evaluate sperm DNA compaction, such as the flowcytometry-based CMA3 and Acrydine Orange, is a reliable strategy to better characterize the sperm chromatin quality in cancer patients at the time of sperm banking and after the initiation of chemotherapy. Because, optimal sperm chromatin packaging seems to be necessary for complete expression of the male fertility potential (77, 78).

O'Flaherty showed that some components of the sperm chromatin structure may recover at different rates after chemotherapy; the significant levels of DNA damage and low chromatin compaction can be detected up to two years after treatment (79). Thus, we consider that it is necessary to screen cancer survivors for the presence of sperm DNA damage before they attempt to achieve fatherhood, either by normal conception or with the aid of artificial reproductive techniques. Moreover, proper counseling for cancer patients at the time of sperm banking and fertility preservation before drug treatment is recommended because of the potential presence of significant sperm DNA damage in the pretreatment semen sample. (Table I)

**Infections**

It has been shown that people with accessory gland infections show leukocytospermia and increase of ROS production (80). On the other hand, leukocytospermic patients have a significant increase in sperm DNA damage compared to normal samples (81). In fact, high level of ROS in these patients, cause to OS, membrane lipid peroxidation along with sperm DNA damage (82). Hepatitis B Virus (HBV) is a serious risk for human health and it can integrate into human sperm chromosomes (83).

Jian-Min Huang *et al* suggested that, HBV infection can lead to modification in genetic component and stimulation of chromosome aberrations and in this way, it causes some genetic abnormalities which may be transmitted to the next generation (84). Another study reported that HBVs exposure can induce ROS production, decrease of total antioxidant capacity, and sperm DNA fragmentation (85). Also, Kang *et al* reported that HBV antigen causes an increase in the sperm DNA fragmentation (85).

Several studies have confirmed that, some types of human papillomavirus cause increase in sperm DNA damage and decrease in sperm motility (86-89). But, another study, didn’t observe any increase of DNA fragmentation index with semen human papillomavirus or Human Herpes Virus infection (90).

In a study, researchers showed that infection showed that infection with several bacteria and fungi such as Escherichia Coli, Chlamydia trachomatis, Ureplasma urealyticum, Staphylococus aureus, Pseudomonas aeruginosa, Candida albicans, and Mycoplasma had a harmful effect on sperm chromatin and sperm DNA integrity (91). In another study, Villegas *et al*, expressed that Escherichia Coli and Staphylococcus aureus, stimulate the expression of apoptosis in human spermatozoa (92). Also, Gallegos *et al*, showed that Chlamydia trachomatis and Mycoplasma cause genitourinary infection and the rate of sperm DNA fragmentation in these patients is higher than non-infected controls (93) (Table I).

**Depression drugs**

Selective serotonin reuptake inhibitors are a class of antidepressants that can affect on the quality of sperm parameters and even lead to sperm DNA damage (94). There is increasing evidence of a relationship between psychological treatments drug and endocrine hormones profile (95). Moreover, several studies have shown that the psychological drugs affect the sperm count, motility and morphology (76, 96). Based on the literature, there is a higher possibility of sperm DNA damage in patients who are under psychological treatment (97, 98) (Table I).

**Alcohol consumption**

Alcohol consumption and its related problems are classified among the top five risk factors for disease, disability and death throughout the world (99). Regarding male infertility, experimental and clinical studies have shown that ethanol consumption alters testosterone secretion, spermatogenesis pattern and Leydig cell volume in addition to inherited aberrant epigenetic signature in offsprings (100, 101). It is reported that ethanol intake in male rats causes a reduction in pregnancy rate and number of pups delivered. This is related to a negative effect of alcohol consumption on normal sperm count, motility and morphology as well as sperm DNA integrity (102-106). Talebi *et al* evaluated the sperm chromatin condensation with cytochemical assays and reported that although the alcohol consumption did not increase the rate of spermatozoa with residual histones and protamine deficiency, it may cause an increase in the percentage of spermatozoa with DNA fragmentation and apoptosis (107).

Another animal study using three different cytochemical tests including CMA3, TB, and SCD showed a negative influence of ethanol on sperm DNA chromatin condensation and fragmentation which was not dose-depended. But, the rate of apoptosis in ethanol-treated mice was higher than controls and it was clearly dose-dependent (108). It is demonstrated that testicular injury by ethanol leads to Fas system up-regulation and increased caspase activity in the testes of ethanol-treated rats which can induce germ cells apoptosis. It can be one of the main causes of maleinfertility associated with alcohol abuse (109). It is shown that chronic alcohol use significantly increased the sperm DNA abnormalities index to 49.6±23.3% compared with 33.9±18.0% in non-drinkers (106). Also, it is revealed that alcohol decreases the levels of DNA methyl transferase transcripts which is the key enzymes in the epigenetic modifications of DNA (110). 

Moreover, both acute and chronic alcohol consumption leads to produce a high level of ROS through the formation of nicotinamide adenine dinucleotide (111). In addition, the products of alcohol metabolism, acetaldehyde, interacts with proteins and lipids to form ROS which affects the plasma membrane and DNA molecules as well as inducing apoptosis in sperm cells (34). Amanvermez *et al* showed that chronic ethanol exposure induces OS in rat kidney, ovary, lung, and testis via high levels of lipid peroxidation and protein oxidation. They also presented that antioxidant supplements can partially defend tissues from ethanol-induced damages formed by ROS (112) (Table I).

**Opiate consumption**

Due to ethical considerations, the studies on the effects of opiate consumption on human fertility are few. However, it is reported that the illicit drugs have deleterious effects on the sperm morphology and motility (113, 114). Albrizio *et al* demonstrated that chronic heroin consumption has been broadly associated to OS, and it causes a significant elevation in DNA fragmentation (115, 116). Also, Song *et al* expressed that ecstasy induces the generation of ROS and reactive nitrogen species and leads to OS (117). 

Experimental data indicate the chronic exposure to ecstasy increases sperm DNA damage, tubular erosion and interstitial edema of testes in male rats (118). One of the most commonly used drugs is marijuana, which causes the release of cannabinoids in the body. Battista *et al* have reported that, these compounds can induce the apoptosis of Sertoli cells and decreases sperm motility, capacitation, acrosome reaction and spermatogenesis (119). 

Another study revealed that, amphetamines lead to a dose-dependent decrease of testosterone and DNA damage (120). Also, Safarinejad *et al* showed that, opiate consumers have a significant increase in the quantity of fragmented DNA in relation to the control group. They also found that the level of catalase-like and superoxide dismutase-like antioxidant activity in opiate consumer were lower and finally, they concluded that illicit drugs have significant adverse effects on the semen quality (121) (Table I).

**Age**

There are some studies which have demonstrated that, increased paternal age is associated with a decrease in sperm morphology, motility and semen volume (122, 123). On the other hand, several studies, have indicated that, there is an association between sperm DNA damage and aging (124, 125). it is shown that sperm nuclear abnormalities such as DNA fragmentation, protamine deficiency and inappropriate chromatin packaging will be increased with age (126, 127).

Wyrobek *et al* showed that young men have a lower percentage of spermatozoa with DNA fragmentation than old men (122). Moskovtsev *et al*, in a retrospective study reported that DNA fragmentation rate in infertile men over 40 years old is higher than infertile men with 40 years old and below (128). ROS are the most powerful inducers of the sperm DNA damage (129, 130) and ROS generation increases with age (131). It is believed that in old men, increasing OS may be responsible for the age-dependent sperm DNA damage and it can be considered as an important etiology of assisted reproductive technique (ART) failures (132). Also, reproductive organs in older men are exposed to OS, for a long time, they may produce more DNA-damaged spermatozoa than youngers (133).

In addition, multiple studies have shown that OS, which tends to increase with age, has deleterious effects on sperm function and quality and may lead to the sperm DNA damage (134, 135). Also, it is indicated that advanced paternal age could lead to an increase in spontaneous abortions (136, 137). Moreover, it has been revealed that increasing paternal age is along with increasing in chromosomal aneuploidies, autosomal dominant disorders and other diseases (138-140). (Table I)

**Lifestyle**

- **Coffee consumption**

Schmid *et al* revealed that men who drink three cups or more caffeine per day have significantly higher rates of sperm DNA damage (138). In fact, Shamsi and Hadi explained that, some of the caffeine products can reduce copper (Cu)(II) into Cu(I), and it leads to production of ROS and in turn, it causes OS (141, 142). On the other hand, caffeine may act as an inhibitor of DNA repair and so, it may be a factor for increasing sperm DNA damage (143).

- **Obesity**

It is almost generally accepted that male obesity is considered as a main risk factor of infertility and it is correlated with a decrease in sperm motility and an increase in sperm DNA damage (144). Kort *et al* by using sperm chromatin structural assay (SCSA) showed that obese patients have a lot of spermatozoa with DNA fragmentation in their ejaculates (145). Tamburrino *et al* suggested that male obesity has negative effects on sperm DNA integrity and fertility (146). In fact, obesity causes an increase in OS and this, in turn is associated with damage to cellular biomolecules such as DNA and fatty acids (147). It should be noted that, there are few studies that they didn’t find any significant correlation between sperm DNA integrity and body mass index but, they used a small sample size in their studies (148, 149).

- **Dietary**

Several studies have shown the effect of antioxidant therapy on sperm parameters (150-152). There are some studies that they have indicated significant progress in sperm concentration, motility and morphology with antioxidant therapy (153). Another study that conducted by Silver *et al* didn’t show any connection between Vit E, Vit C, β-carotene therapy and the sperm DNA damage (154). Alcohol consumption, cigarette smoking and dieting cause to vitamin and antioxidant deficiency which it can lead to oxidative damage (155-157).

- **Occupation **

It has been shown the occupational exposure such as styrene can change the integrity of DNA in male germ cells (158). Many chemical materials affect the reproductive system (glands and hormones) (159). A study has shown that polychlorinated biphenyls (PCBs) can decrease the sperm parameters and pregnancy outcome (160). Also there are several studies that they have reported the negative effects of chemical pesticides on infertility (161-163).

- **Smoking**

The exact effect of smoking on male fertility has remained controversial in the literature. Harmful substances like alkaloids, nitrosamines, nicotine and inorganic molecules are present in tobacco and cigarettes that produce reactive oxygen/nitrogen species (164, 165). There is a significant association between active smoking and reduced seminal quality, sperm DNA integrity and nuclear maturation, increased sperm DNA fragmentation and induced alterations of the sperm plasma membrane (166-170). High levels of DNA strand breaks in smoker men may be due to the presences of carcinogens and mutagens in cigarette smoke (171). In a study performed on 655 smokers and 1131 non-smokers, a significant decrease was shown in sperm density (15.3%) as well as other parameters including total sperm count (17.5%), and total number of motile spermatozoa (16.6%) (172). Therefore, smoking may have detrimental effects on the quality and quantity of sperm in male and decreases the antioxidant activity in seminal plasma (173).

Saleh *et al* demonstrated increasing (approximately 48%) in seminal leukocyte concentrations and ROS levels in infertile smoker men when compared with non-smokers. They concluded that there may be a positive association between leukocytospermia and ROS levels (166). Potts *et al* also investigated sperm DNA damages of 35 fertile smokers and 35 fertile nonsmokers with SCSA and reported the higher sperm DNA damage in smokers compared to nonsmokers (174). Several investigators have shown a correlation between cigarette smoking, OS and sperm DNA damage (94, 148-150). Regard to sperm chromatin, the results have suggested that the induction of OS by cigarette smoking may negatively affect the protamination process by disrupting protamine 2 in sperm chromatin (175-177). On the other hand, it is shown that cigarette smoking can suppress the function of miRNAs that play a crucial role in regulating gene expression and epigenetic patterns associated to spermatogenesis and sperm chromatin structure (178, 179).

- **Cellphone**

Today, there are multiple studies that focus on the effect of electronic systems such as mobile, telephones, televisions and microwaves on the reproductive system. it has been reported theabnormal semen parameters and defect in acrosomal reaction along with the use of cellphone (180-184). Recent studies have shown that Radio-frequency electromagnetic radiation, of mobile phones, may cause to increase of mitochondrial ROS production and DNA fragmentation (185-187). So, it is believed that extensive mobile phone use by men may affect male fertility potential and even the health of their offspring (Table I).

- **Air pollution**

Over the past decades, many studies have shown that environmental pollution is implicated in poor sperm quality (188, 189). There is some evidence of increased percentage of spermatozoa with chromatin abnormality with fragmented DNA in selected population like motorway workers because of prolonged exposure. However, there was no significant relationship between blood concentrations of Pb, NO_2_ or SO_2_ and sperm chromatin and/or DNA damage. Serum levels of LH, FSH, and T did not differ significantly, whereas the sperm parameters were significantly different in motorway tollgate workers (190). 

In another selected examined sample study, likely, much higher levels of sperm DNA damage were seen in men following periods of high air pollution from burning of coal, with no obvious change in other semen parameters (191). Ultimately, it could be hypothesized that a combination of pollutants would be responsible for sperm abnormalities, rather than a single heavy metal (190). 

Some findings provided evidence that polymorphisms of some genes may be useful biomarkers to identify individuals susceptible to DNA damage. XRCC1 Polymorphisms may alter sperm polycyclic aromatic hydrocarbon-DNA (PAH DNA) levels and could be considered as the useful biomarkers to detect individual susceptibility to DNA damage resulting from exposure to PAHs (192). Polymorphisms of the DNA repair genes XPD6 and XPD23 and a polymorphism CYP1A1MspI as a metabolic gene were associated with high levels of sperm DNA fragmentation in men with a high exposure level of air pollution (193). Evidencesuggest that men will respond in a different manner to the same level of exposure and that individual responses may be modified by different level of gene expression (194).

**High temperature**

The High temperature is a physical factor which is concerned about for decades. It can affect the male reproductive system either directly, causing decreased or altered sperm production, or indirectly through the endocrine system causing a hormonal imbalance (195). Data from experimental studies showed scrotal heating due to the posture of sleeping and sitting, driving and clothing may affect testicular function and is related to male infertility (196-198). However, making the selection of an appropriate control group is difficult or impossible and current data is not available from human sperm DNA quality in response to heat. However, in an experimental study, when mice were exposed to increased temperature, a stress-induced apoptosis in epididymis and testis which are very functional was seen. Germ cells in the testis were either lost by apoptosis or went on to complete their developmental process and were recovered as motile spermatozoa with fragmented DNA (199).

## Conclusion

In highly compacted toroidal nucleo-protamine complexes, there is a nonrandom distribution of genes have potential to be involved in the genomic activation in the early embryo. In addition, protamines and other sperm chromatin associated proteins may provide epigenetic information to serve the reorganization of paternal chromatin after fertilization. Meanwhile, DNA strand breaks delay the replication until either the damage can be repaired or until embryo development with damaged DNA is no longer probable. 

An overall consideration of the current record on human sperm chromatin/DNA shows that environmental, clinical and iatrogenic relevant factors might reflect dysmetabolism. The consequence of dysmetabolism is endocrine disrupting compounds which alter remodeling of sperm chromatin which can be detected in infertile patients. Although SCSA is considered as the gold standard and other detecting methods of individual sperm such as SCD, CMA3, TB, and AB are preferred for their simplicity, this should be noted that post meiotic damage may regularly occur in the sperm DNA and a prognostic test to help as a true screening test to appraise DNA integrity of motile and non-apoptotic fraction of the sperm population have not been achieved.

## Conflict of interest

All investigators disclose no conflict of interest in this study.
